# Effects of Vegetation Phenology on Ecosystem Water Use Efficiency in a Semiarid Region of Northern China

**DOI:** 10.3389/fpls.2022.945582

**Published:** 2022-07-04

**Authors:** Yaru Zhang, Jing Zhang, Jianyang Xia, Yahui Guo, Yongshuo H. Fu

**Affiliations:** ^1^College of Water Sciences, Beijing Normal University, Beijing, China; ^2^School of Ecological and Environmental Sciences, East China Normal University, Shanghai, China

**Keywords:** water use efficiency, phenology, climate change, carbon-water cycle, gross primarily productivity (GPP), evapotranspiration (ET)

## Abstract

Water use efficiency (WUE) is an important ecosystem functional property for measuring coupled relationships of the carbon-water cycle. Both biotic and environmental factors significantly impact WUE in terrestrial ecosystems. Relationships between environmental factors and WUE have been well discussed in previous studies. Although the crucial role of vegetation phenology, a common indicator of climate-vegetation interactions, in regulating the WUE has been widely reported, the underlying mechanism has not yet to be fully elucidated. Here, we utilized multiple long-term remote sensing datasets to analyze the interannual changes in seasonal WUE, and discussed the potential associations between phenology and WUE in the Luanhe River basin, which is a typical semiarid region of China, from 1988 to 2015. Most of the pixels across Luanhe River basin showed increasing spring WUE and decreasing autumn WUE. The start of the growing season (SOS) was slightly advanced by an average of 0.06 days per year (d/y) in the whole study area, with a delayed trend (0.04 d/y) in the upper reaches region (UR) and an advanced trend (0.20 d/y) in the middle-lower reaches region (MLR). The end of the growing season (EOS) showed a generally delayed trend (0.11 d/y) across the basin. Furthermore, negative correlations were detected between spring WUE and SOS in the UR, while positive correlations were detected in the MLR. The opposite patterns of the relationship of autumn WUE and EOS were also found between UR and MLR. The results were attributed to the balance and compensation of biotic and abiotic factors in the regulation of ecosystem WUE. Our findings provide new insights into the interaction between vegetation dynamics and carbon-water cycle coupling.

## Introduction

Climate change profoundly impacts carbon-water cycles by controlling the carbon sequestration capacity and water resource allocation in terrestrial ecosystems (Ponce Campos et al., 2013; Wang et al., 2021). Water use efficiency (WUE), the process of carbon-water cycle coupling, reflects the efficiency of plants in using water to produce dry matter and is controlled by physiological and physical processes (Le Houerou, 1984; Lu et al., 2017; Cheng et al., 2021). Phenology is widely considered the most sensitive indicator of climate change (Piao et al., 2010, 2019; Fu et al., 2015). Recent climate warming has extended the growing season mainly due to the advanced start of the season (SOS) and the delayed end of the season (EOS) in the Northern Hemisphere (Menzel et al., 2006; Peñuelas, 2009; Geng et al., 2020), which has subsequently affected the carbon-water cycle. However, it has not been well investigated how phenological shifts affect the regional carbon-water cycle coupling process, especially at the watershed scale. Hence, understanding the spatial-temporal dynamics of WUE and its response to climate change is essential for obtaining insight into vegetation water use strategies and then clarifying the adaptability of the ecosystem to climate change (Yang et al., 2016; Tarin et al., 2020).

The relationships of ecosystem WUE and meteorological factors have been widely documented, and they vary greatly under different climate conditions. For example, both increased (Huang et al., 2016; Zhang et al., 2019) and decreased (De Boeck et al., 2006; Bell et al., 2010) WUE in response to increased temperature have been reported. With the increase in precipitation, increased WUE was found in drier regions, whereas decreased WUE was found in wetter regions (Tian et al., 2010; Huang et al., 2016; Zhou et al., 2017). Vapor pressure deficit (VPD), one of the major drivers, significantly affects WUE in rainfed maize croplands (Lan et al., 2021). However, the impact of VPD on WUE was insignificant in the tropical rainforest ecosystem (Aguilos et al., 2018). The mechanisms underlying diverse responses of WUE to changes in meteorological factors remain unclear.

In addition to meteorological factors, biophysical factors also significantly affect the carbon-water cycle of terrestrial ecosystems. As the most sensitive indicator of climate-vegetation interactions, phenology plays an important role in the regulation of WUE. Variability in phenology leads to changes in the growing season. It is generally investigated whether the extension of the growing season enhances light interception and prolongs photosynthetic activity, thereby increasing ecosystem gross primary productivity (GPP) (White et al., 1999; Guo et al., 2020). Meanwhile, plant transpiration increases, whereas soil evaporation decreases, resulting in variation in evapotranspiration (ET) (Liu et al., 2018; Guo et al., 2020). The changes in GPP and ET further result in the interannual variability in WUE (Beer et al., 2009; Xiao et al., 2013; Yang et al., 2016). However, the impacts of changes in SOS and EOS on WUE are quite different, yet these differences are rarely studied and involve many uncertainties. Furthermore, given the significant differences in climatic conditions and vegetation types among different study areas, the variation in WUE has been highly spatially inconsistent in response to climate change. To our knowledge, how the phenological shift affects seasonal WUE under different climatic conditions, especially hydrothermal conditions, has not been well investigated. Hence, it is essential to integrate meteorological and phenological factors to explore the spatiotemporal changes and mechanisms of seasonal WUE.

Considering the significance of vegetation phenology and climate change on WUE, we focused on the Luanhe River basin, a typical semiarid watershed in temperate China. The whole river basin was separated into two subregions by local land-cover types and climatic conditions (Geng et al., 2020). The upper reaches region (UR) is drier and mainly covered by temperate and meadow grassland, whereas the middle-lower reaches region (MLR) is wetter and mainly covered by temperate forests. Previous studies have reported that phenology has changed significantly with large spatial heterogeneity in the past three decades (Geng et al., 2020). Using long-term remote sensing-based GPP and ET and meteorological and normalized difference vegetation index (NDVI) datasets, the spatiotemporal changes in WUE were studied in the Luanhe River basin from 1988 to 2015. Specifically, our objectives were to answer the following questions: (1) How did the WUE, GPP, and ET change during 1988-2015 in the Luanhe River basin? (2) What are the relationships between WUE, SOS, and EOS? Are there seasonal differences in the relationships between phenology and WUE? (3) What are the driving mechanisms behind the interannual changes in seasonal WUE?

## Materials and Methods

### Study Area

The Luanhe River basin, a typical temperate continental monsoon region, is located in the arid and semiarid areas of North China (40°00′N to 42°41′N, 115°27′E to 118°56′E). The total area is 38,000 km^2^ and includes 12 main sub-basins, with high elevations in the northwest and low elevations in the southeast. Due to the large latitude span and the elevation difference, the natural geographical conditions vary significantly from arid and semiarid climates in the northwest to semi-humid climates in the southeast. The natural vegetation is mainly grassland in the northwest and forests in the southeast. According to the distribution of land covers in 2015, the Luanhe River basin is divided into two regions: the upper reaches region (UR, mainly covered by grassland) and the middle-lower reaches region (MLR, mainly covered by temperate forests) (Geng et al., 2020). As one of the most sensitive areas to climate change, the Luanhe River basin is facing great climatic and ecological risks. Furthermore, serving as one of the most important water systems in North China, the Luanhe River basin is the forefront barrier in the Beijing-Tianjin-Hebei metropolitan region in China.

### Data and Processing

#### Phenology Datasets

The SOS and the EOS were used as the proxies of vegetation growth in this study. These long-term phenological records were extracted from the bimonthly 8-km Global Inventory Modeling and Mapping Studies (GIMMS) NDVI3g (third-generation normalized difference vegetation index) dataset from the Advanced Very High Resolution Radiometer (AVHRR) (Tucker et al., 1994). Five commonly used methods (the Gaussian-midpoint, spline-midpoint, HANTS-maximum, polyfit-maximum, and Timesat-SG methods) were applied to determine the SOS and EOS. There were three steps for extracting the phenological dates: (1) eliminate the influence of noise and errors caused by orbital drift, calibration, observation geometry, stratospheric volcanic aerosol and snow cover to get a smooth NDVI curve; (2) filter and interpolate the NDVI curve, and use the threshold or the maximum/minimum rate of time NDVI change to determine the natural date; (3) identify and remove the abnormal points based on five-point median-value moving average method. The mean SOS and EOS across the five methods were applied for each pixel and subsequent calculations and analyses.

#### Gross Primary Productivity and ET Data

Remote sensing data have been widely used due to their long-time span and wide range in the study of ecosystem WUE. Vegetation Optical Depth Climate Archive Version 2 Gross primary productivity (VODCA2GPP)^1^ with a 0.25° spatial resolution and 8-day temporal resolution from 1988 to 2015 was used in this study, and the unit of GPP was gC m^–2^ d^–1^ (Wild et al., 2021). Vegetation optical depth (VOD) describes the radiation attenuation of vegetation in the microwave domain, which is controlled by its water content, biomass, type, and density (Mo et al., 1982; Vreugdenhil et al., 2016). Since VOD is sensitive and positively correlated to vegetation growth dynamics, meanwhile, VOD is less affected by weather conditions, it is usually used to retrieve vegetation growth status and monitor vegetation biomass. The dataset we used in this analysis, i.e., VODCA2GPP, derived GPP from VOD using the carbon sink driven method. Compared with the most commonly used GPP inversion methods based on light energy utilization efficiency and optical remote sensing variables (such as MODIS GPP, GLASS GPP), VODCA2GPP is less affected by weather conditions, thus is more accurate and realistic with longer period. According to the introduction of VODCA2GPP, the dataset combined VOD observation data from multiple sensors and has been evaluated and verified by flux stations and other independent GPP datasets, including MODIS GPP, FLUXCOM GPP, as well as GPP data sets simulated by TRENDY-v7 model. The evaluation and verification results show that, compared with the existing GPP data sets, VODCA2GPP is closely related to MODIS GPP and TRENDY-v7 GPP (Pearson correlation coefficients were 0.53 and 0.61, respectively) with highly consistent fluctuation in long time series and lowest uncertainty. Therefore, the VODCA2GPP can ensure the accuracy and reliability required by the study.

A high-resolution data-oriented monthly ET product^2^ in China was used with 0.1° spatial resolution and 30 days temporal resolution (Li et al., 2018). Using the observation date of 36 flux tower stations in China, this product was developed by integrating remote sensing and the eddy covariance technique to observe ET data by the machine learning approach (model tree ensemble, MTE), which has been proven to be robust in extrapolating ET to regions not covered by eddy covariance towers (Jung et al., 2010). Compared with the complex models, MTE method can scientifically and reasonably extrapolate the measured value of ET to the area without flux tower, and connect ET with various environmental and climate driving variables. Thus, it is a supplement to the process-based model. Compared with the existing ET data set, it is found that this dataset included more measured data to eliminate the error caused by solar radiation. In addition, the ET dataset in China has higher spatial resolution and longer time period, which can better meet the accuracy and scientific requirements of this study.

The GPP dataset was scaled to match the spatiotemporal resolution of the ET dataset.

The ecosystem WUE indicator used in this study was calculated as follows:


(1) $$\text{WUE}=\frac{\text{GPP}}{\text{ET}}$$

where GPP is the gross primary productivity (gC m^–2^ d^–1^) and ET is the evapotranspiration (kg H_2_O m^–2^ d^–1^).

#### Meteorological Data

Total precipitation (mm) and mean air temperature (°C) with a spatial resolution of 0.1° from 1988 to 2015 were extracted from the China Meteorological Forcing Dataset (CMFD) (He et al., 2020).^2^ Based on the daily data of these climatic factors, the seasonal mean precipitation and temperature were calculated and used to obtain the mechanism affecting the variation in WUE. Monthly temperature and actual vapor pressure (AVP) were collected for the calculation of vapor pressure deficit (VPD). According to the improved Magnus empirical formula recommended by the China Meteorological Administration,^3^ the VPD equations are as follows:


(2) $$\text{SVP}=610.78\times \text{exp}\,\left[\frac{17.269\,\left(T-273.16\right)}{T-35.86}\right]$$

(3) VPD=SVP-AVP

where T is the water (ice) surface temperature, which is usually replaced by the mean air temperature. SVP represents the saturated vapor pressure, and AVP is the AVP. VPD was calculated by SVP and VAP by Equation (3).

We aggregated every variable type of data to a monthly scale for further analysis and then calculated the seasonal average value. The seasons in this analysis were spring and autumn, defined as April–May and September-October, respectively.

### Statistical Analysis

Before analysis, all original datasets were interpolated and reconstructed into 0.1°×0.1°using nearest neighbor interpolation. The linear regression slope between the time series and every variable series represents the trend of seasonal WUE, GPP, ET, phenology, and climatic factors. Furthermore, we normalized the GPP, ET and WUE series using min-max normalization and analyzed the sensitivity of WUE to GPP and ET, respectively. The sensitivities reflect the change in WUE per unit change in GPP or ET. After that, to assess the impacts of SOS and EOS on the interannual variation in spring and autumn WUE, the partial correlation coefficients between the time series of phenology, WUE, and climatic factors (temperature, precipitation, VPD) were calculated for every pixel. The greater the correlation coefficients, the stronger the dominance. In addition, to distinguish the spatial-temporal difference in the WUE response to vegetation phenology, all the correlation coefficients were analyzed in the two subregions (i.e., UR and MLR) and two seasons (i.e., spring and autumn), respectively. Then, combined with the calculation method of WUE, we took the meteorological and phenological factors as the first-order variables, and took GPP and ET as the second-order variables to build the structural equation model (SEM). SEM was used to explore the regulatory mechanisms of meteorological and biophysical factors for seasonal WUE. SEM is a statistical method to analyze the relationship between variables based on the covariance matrix. The hypothetical model was constructed based on theoretical and empirical knowledge, and the reasonable path coefficient was determined based on statistical methods. Therefore, SEM can solve the causal relationship and distinguish the direct and indirect effects that cannot be obtained in correlation analysis. Using the SEM program AMOS (version 21.0, IBM SPSS, Chicago, Illinois), one of the most standard software programs of the SEM methodology (Romero-Rodriguez et al., 2020), we constructed the cascading effect networks of climatic and phenological factors–GPP and ET–WUE, and the effect of one variable on another variable can be quantified by the standardized path coefficient (ρ).

## Results

### Changes in Water Use Efficiency, Gross Primary Productivity, and ET in Spring and Autumn

**FIGURE 1:**
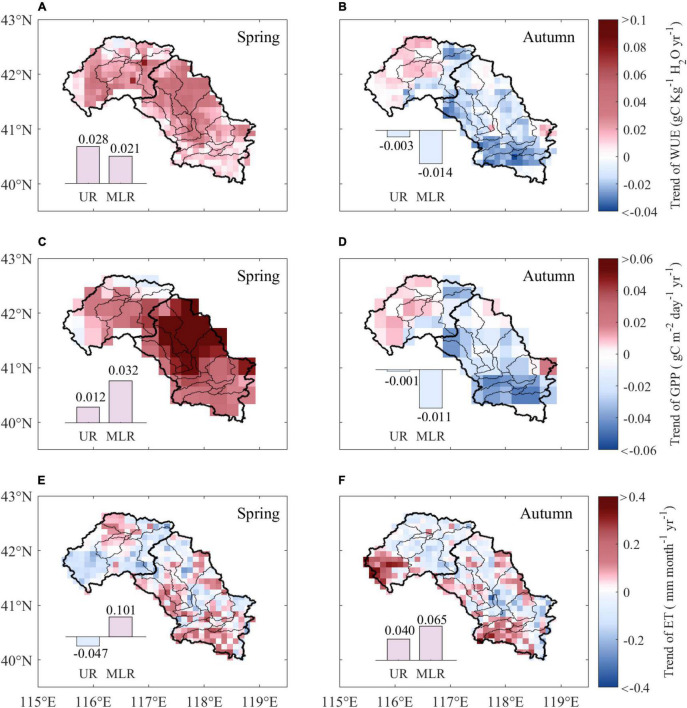
Trends of WUE, GPP, and ET from 1988 to 2015 during spring and autumn. The subpanels show the difference in trends of WUE **(A,B)**, GPP **(C,D)**, and ET **(E,F)** between the UR and MLR. UR is the upper reaches region, and MLR is the middle-lower reaches region.

The linear regression results for WUE showed an overall increasing trend during spring across the Luanhe River basin, and the average trend was 0.024 gC kg^–1^ H_2_O per year, with approximately 35.6% of the study area being statistically significant (*p* < 0.05). Although increasing trends of WUE existed in both the UR and MLR, the increase in WUE in the UR (0.028 gC kg^–1^ H_2_O yr^–1^) was greater than that in the MLR (0.021 gC kg^–1^ H_2_O yr^–1^) (Figure 1A). The mean annual trend of WUE during autumn was −0.006 gC kg^–1^ H_2_O yr^–1^ over the whole area. Large spatial differences in WUE changes existed during autumn, and the decreasing WUE was mainly observed in the MLR (−0.014 gC kg^–1^ H_2_O yr^–1^), whereas the WUE in the UR (−0.003 gC kg^–1^ H_2_O yr^–1^) was much smaller (Figure 1B).

The spatial distribution of the annual trend of GPP is shown in Figures 1C,D. The GPP showed an overall increasing trend across the study area during spring, with an average trend of 0.027 gC m^–2^ day^–1^ per year, 54.5% of which was significant (*p* < 0.05). The mean increasing trend of spring GPP in the MLR (0.032 gC m^–2^ day^–1^ yr^–1^) was more than twice that in the UR (0.012 gC m^–2^ day^–1^ yr^–1^) (Figure 1C). However, the changes in GPP during autumn showed distinct spatial distributions. Approximately 69.8% of pixels on GPP experienced decreasing trends and were mainly observed in MLR (−0.011 gC m^–2^ day^–1^ yr^–1^), yet there was almost no change in GPP in UR during autumn (−0.001 gC m^–2^ day^–1^ yr^–1^) (Figure 1D).

The spatial distribution of annual changes in ET was of higher heterogeneity. The diversity among pixels was greater, lacking watershed unified spatial differences. The ET trend varied from −0.255 to 0.262 mm month^–1^ year^–1^ with a mean of −0.013 mm month^–1^ year^–1^ in the Luanhe River basin during spring, with only 2.16% of the study area being significant (*p* < 0.05). Large spatial differences were detected in the UR and MLR; ET decreased with a trend of −0.047 mm month^–1^ year^–1^ in the UR and increased with a trend of 0.101 mm month^–1^ year^–1^ in the MLR (Figure 1E). Compared to the results of spring, the autumn ET showed similar trends in UR and MLR, with average increasing trends of 0.040 mm month^–1^ yr^–1^ and 0.065 mm month^–1^ yr^–1^, respectively (Figure 1F).

### Key Factors Affecting the Changes of Water Use Efficiency

**FIGURE 2:**
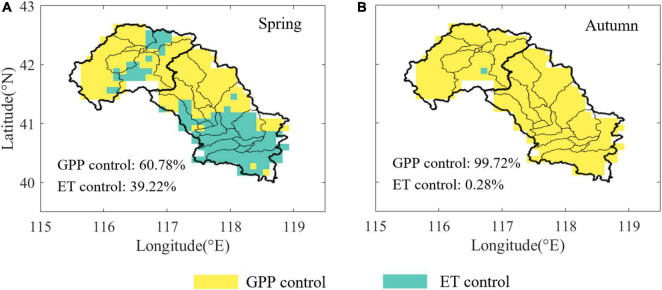
Distribution of normalized key factors affecting WUE across the Luanhe River basin. **(A)** The distribution during spring **(A)** and during autumn **(B)**. The color indicates that the WUE change of each pixel is mainly controlled by GPP (yellow) or GPP (green).

Interesting patterns were observed for key factors affecting the temporal changes in WUE, probably reflecting differences in the leading role of the carbon-water cycle in different ecosystems and seasons. The dominant driving factor of WUE during spring was GPP, which occupied 60.78% of the study area (Figure 2A). ET played a dominant role mainly in the southern part of the MLR, which led to great differences in the distribution of dominant factors between the UR and MLR. During autumn, GPP served as the dominant driver affecting the variation in WUE in 99.72% of pixels in the Luanhe River basin, which was highly consistent in both the UR and the MLR (Figure 2B).

### Changes of Phenology and Climate Variables

#### Phenological Dynamics

**FIGURE 3:**
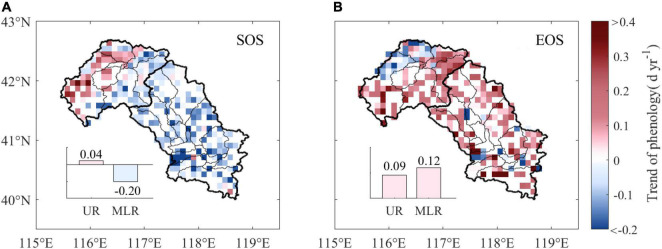
Annual trends of phenology from 1988 to 2015. The subpanels show the difference in annual trends of the SOS **(A)** and EOS **(B)** between the UR and MLR. UR is the upper reaches region, and MLR is the middle-lower reaches region.

The SOS advanced by an average of 0.06 days per year (d yr^–1^) in the Luanhe River basin over the period 1988–2015, and approximately 73.8% of the study area experienced advanced trends, with 22.5% of them being statistically significant (*p* < 0.05). A total of 26.2% of the pixels experienced delayed trends and were mainly distributed in the UR, where the average trend of the SOS was 0.04 d yr^–1^. However, the SOS advanced with an average rate of 0.20 d yr^–1^ in the MLR (Figure 3A).

The mean EOS trend was 0.11 d yr^–1^ over the Luanhe River basin during 1988–2015. A delayed trend was observed in 83.4% of the study area, of which 51.2% was significant (*p* < 0.05). The EOS trends between the UR and MLR were slightly different. The delayed EOS in the UR (0.09 d yr^–1^) was slightly smaller than that in the MLR (0.12 d yr^–1^) (Figure 3B).

#### Climatic Dynamics

**FIGURE 4:**
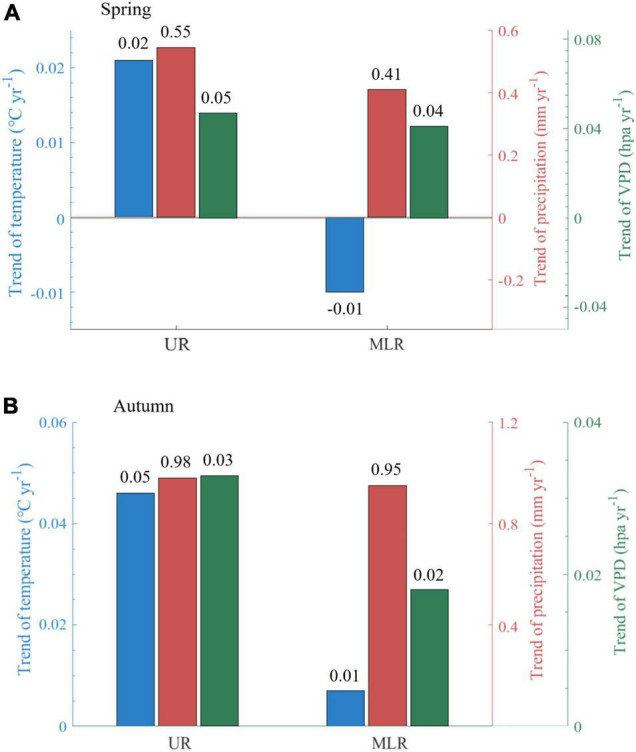
Annual trends of climatic factors (temperature, precipitation, VPD). The subpanels show the difference in spring **(A)** and autumn **(B)**. UR is the upper reaches region, and MLR is the middle-lower reaches region.

During 1988-2015, it became warmer and wetter in spring in the UR of the Luanhe River basin, with the daily mean air temperature increasing by 0.02^°^C per year (^°^C yr^–1^), the total precipitation increasing by 0.55 mm per year (mm yr^–1^), and the VPD increasing by 0.05 hPa per year (hPa yr^–1^). However, the MLR of the Luanhe River basin became colder in spring by −0.01^°^C yr^–1^, and the changes in total precipitation and VPD were smaller than those in the UR. The total precipitation increased by 0.41 mm yr^–1^, and the VPD increased at a rate of 0.04 hPa yr^–1^ (Figure 4A). Significant differences were found in daily mean air temperature between UR and MLR.

Trends of meteorological factors in autumn are relatively consistent in the UR and MLR, but their rates were lower in the MLR than in the UR. The daily mean air temperature was increased by 0.05^°^C yr^–1^ in the UR, approximately five times that in the MLR (0.01^°^C yr^–1^). The total precipitation increased by 0.98 mm yr^–1^ in the UR and 0.95 mm yr^–1^ in the MLR. The increase in VPD was stronger in the UR than in the MLR, with averages of 0.03 hPa yr^–1^ and 0.02 hPa yr^–1^, respectively (Figure 4B). Significant differences were found in both temperature and VPD between UR and MLR.

### Correlations Between Phenology and Water Use Efficiency

**FIGURE 5:**
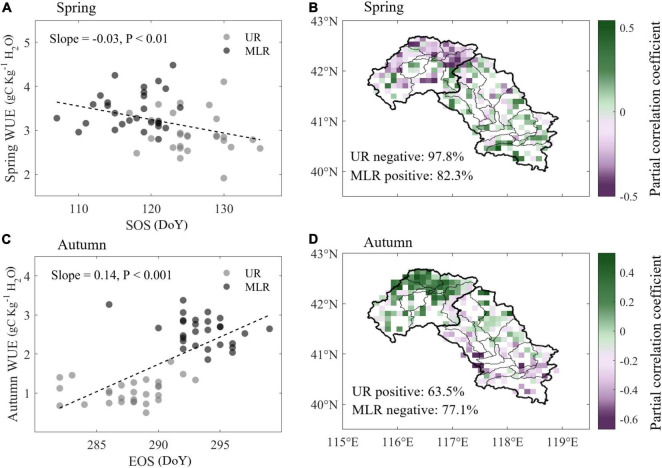
Correlations between WUE and phenology during spring and autumn. **(A,C)** Linear regression of annual WUE and phenological dates in the UR and MLR, respectively. **(B,D)** Spatial distribution of partial correlation coefficients between WUE and phenology.

Using linear regression between spring WUE and SOS, we found that with the delay of SOS, spring WUE decreased by 0.03 gC kg^–1^ H_2_O d^–1^ across Luanhe River basin (Figure 5A). When divided into two parts, we found that WUE decreased by −0.01 gC kg^–1^ H_2_O d^–1^ in the UR, whereas WUE increased by 0.03 gC kg^–1^ H_2_O d^–1^ in the MLR. Converse relationships between WUE and SOS in UR and MLR were also detected. According to the spatial distribution of the partial correlation efficiency between WUE and SOS, WUE was negatively correlated with SOS in 97.8% of the UR area but was positively correlated with SOS in 82.3% of the MLR area (Figure 5B).

In autumn, WUE showed significantly increase with the delay of EOS by 0.14 gC kg^–1^ H_2_O d^–1^ (Figure 5C). Furthermore, with the delay of EOS, WUE was significantly increased by 0.04 gC kg^–1^ H_2_O d^–1^ in the UR but decreased by −0.01 gC kg^–1^ H_2_O d^–1^ in the MLR, although the increase was insignificant. The conversely spatial pattern was also detected in autumn (Figure 5D). Autumn WUE was positively correlated with EOS in 63.5% of the UR but negatively correlated with EOS in 77.1% of the MLR.

### Cascading Relationships of Factors Influencing Spatial Variations in Water Use Efficiency

The SEM results indicated that climatic factors (daily air temperature, sum precipitation, VPD) and biological rhythm factors (SOS and EOS) jointly determined the variation in WUE by influencing GPP and ET due to the cascading effects of climatic factors and phenological factors on WUE. In addition, it was noted that the path coefficients and relative importance of all factors affecting WUE were different in the Luanhe River basin.

**FIGURE 6:**
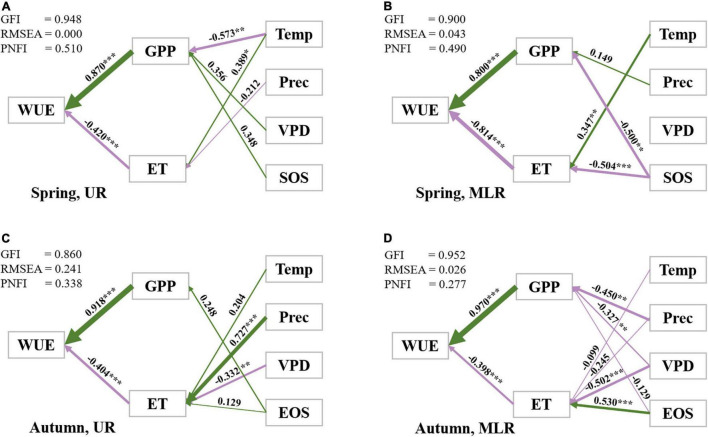
Path diagrams of the interannual variations in WUE in the UR **(A,C)** and MLR **(B,D)**. Standardized path coefficients (ρ: −1 to 1) are listed along with path arrows, of which the thickness was consistent with the regression weights. ρ > 0 indicates positive correlations along with green arrows, while ρ < 0 indicates negative correlations along with purple arrows. Significant (*p* ≤ 0.05) paths and insignificant (*p* > 0.05) paths were distinguished with *. *** indicates *p* ≤ 0.001, ** indicates 0.001 < *p* ≤ 0.01, *** indicates 0.01 < *p* ≤ 0.05.

For the UR during spring, GPP had the strongest direct effect (ρ_WUE–GPP_ = 0.870) on WUE, while ET had the opposite direct effect (ρ_WUE–ET_ = −0.420), which partly offset the impacts of GPP. Temperature served as an important indirect effect on WUE by mainly influencing GPP (ρ_GPP–Temp_ = −0.573) and ET (ρ_ET–Temp_ = 0.389) (Figure 6A).

For the MLR during spring, both GPP and ET had nearly equivalent strong direct effects on WUE (ρ_WUE–GPP_ = 0.800, ρ_WUE–ET_ = −0.814, respectively). SOS had significant negative effects on GPP and ET, which indirectly led to the interannual variation in WUE. The daily mean air temperature was another driving factor of ET and had a significantly positive effect on ET (ρ_ET–Temp_ = 0.347) (Figure 6B).

For the UR during autumn, GPP was the most important controlling factor of WUE (ρ_WUE–GPP_ = 0.918) compared with ET (ρ_WUE–ET_ = −0.404). Among all climatic factors, precipitation and VPD had the largest impacts on ET (ρ_ET–Prec_ = 0.727, ρ_ET–VPD_ = −0.332, respectively), while other factors were relatively less important (Figure 6C).

For MLR during autumn, GPP was the dominant driving factor with a coefficient of 0.970 and was more than twice ET on WUE. Unlike what was observed in the UR, precipitation had a negative correlation with GPP (ρ_GPP–Prec_ = −0.450), and VPD had negative correlations with both GPP (ρ_GPP–VPD_ = −0.327) and ET (ρ_ET–VPD_ = −0.502) but a higher correlation with ET. In addition, EOS played the predominant role in regulating the interannual variation in ET among all climatic factors (ρ_ET–EOS_ = 0.530) (Figure 6D).

## Discussion

### Phenological Shifts and Spatial Distribution

Average advanced SOS and delayed EOS were found across the Luanhe River basin over the period 1988–2015, which is consistent with previous studies (Cong et al., 2012; Zhou et al., 2020). The reverse changes in SOS and EOS mainly contributed to the cascading effects of thermal and hydrological conditions, but the mechanisms differed among regions (Richardson et al., 2013; Wang et al., 2017). SOS was delayed in the UR, where grasslands are mainly covered. Phenological shifts are more sensitive to changes in precipitation or soil moisture in grasslands (Chuai et al., 2013). Therefore, warmer winters lead to less precipitation and lower soil water content, resulting in delayed SOS in the UR. However, the SOS was mainly controlled by thermal conditions in the MLR, where forests are the dominant biome. Warmer winter and spring promote spring phenology (Huang et al., 2016). In addition, other climatic factors, such as photoperiod, also affect phenological dynamics (Fu et al., 2019), especially under ongoing global warming. However, how the photoperiod affects the vegetation phenology between grasslands and forests is unclear, and further investigations are thus proposed.

### Interannual Variability of Water Use Efficiency

Over the Luanhe River basin, significant differences were found in the trend of WUE between spring and autumn. An overall increasing trend was detected in spring, while a mainly decreasing trend was shown during autumn. Moreover, the decreasing magnitude of autumn WUE in the MLR was much greater than that in the UR. Guo et al. (2020) investigated the variation in seasonal WUE in subtropical forests in Zhejiang Province, China, from 2000 to 2014, and found similar results: spring WUE exhibited an overall increasing trend, while autumn WUE exhibited a large-scale decreasing trend. Our results thus support that opposite patterns in WUE variability between spring and autumn and further demonstrates different roles of SOS and EOS in the regulation of seasonal WUE at the watershed scale.

In spring, WUE and SOS were negatively correlated in the UR. An earlier SOS leads to an earlier start of photosynthesis and higher vegetation growth (Geng et al., 2020), resulting in a rapid increase in GPP (Luyssaert et al., 2007). Meanwhile, earlier leaf expansion or a larger leaf area increased plant transpiration but reduced soil evaporation; therefore, the ET changes were not significant in the UR (Beer et al., 2009; Von Arx et al., 2012; Shen et al., 2015). Considering the dominant contribution of GPP to the changes in WUE, spring WUE thus increased with the advancement of the SOS. In the MLR, where forests are mainly covered, the increase in WUE was affected by the combined effects of GPP and ET. Forest-covered areas usually have higher water interception and transpiration than grassland-covered areas (James et al., 2003; Yamazaki et al., 2004). With the advancement of SOS, earlier leaf expansion and larger leaf area enhanced GPP and plant transpiration, even playing a more vital role in increasing ET. Therefore, the correlations between WUE and SOS in the UR and MLR were opposite.

In autumn, delayed EOS leads to a more extended extra photosynthetic period, which is responsible for the increase in GPP. Together with the dominant driving role of GPP in autumn WUE in the UR, WUE was thus positively correlated with EOS. However, EOS was closely correlated with ET due to the water interception function of forest vegetation. GPP was the dominant driver of changes in WUE in the MLR during autumn; therefore, the correlation between WUE and EOS was not that significant.

### Uncertainties

Previous studies have reported that phenology is a key factor impacting terrestrial hydrological cycles and responses under climate change (Nohara et al., 2006; Ragettli et al., 2016). Consistent with these studies, it was found that only climatic factors could not explain the variations in WUE, and the association between phenology and WUE should be valued. Moreover, the correlations between WUE and phenological events depend on climatic conditions and land cover types. Through the cascade relationships, phenology mainly regulated the WUE variations in the MLR, where forests are mainly present, rather than in the UR, where grasslands are primarily present. The interannual changes in WUE in the UR were mainly controlled by meteorological factors. The differences might be attributed to the different vegetation types. According to the key phenological dates extracted from NDVI, SOS mainly occurred in April or May. However, the field survey reported that forbs such as *Potentilla acaulis* L. start their growing season at that time as the dominant species, which accounts for a very small proportion of productivity in the grassland ecosystem due to their physiological structure and morphological characteristics. The grasses [such as *Agropyron cristatum* (L.) *Gaertn*.], which are the main forces of grassland productivity, start their growing season in June and begin to senesce after several rain events in September. Hence, the critical phenological dates attracted from NDVI may do not represent the actual growth stage of dominant species contributing to ecosystem GPP, which can explain the detected minor impacts of phenology on WUE in the UR. It is worth noting that remote sensing datasets might bring uncertainties, so field surveys and observation datasets are necessary to minimize the inaccuracy.

For the MLR, which is covered by forests, the deep root system and its higher capability of resistance to drought maintain the WUE response to meteorological factors in a less obvious manner compared to grassland. Meanwhile, phenological changes directly regulate the effective photoperiod and leaf area, further affecting the ecosystem GPP and ET, resulting in the variation of WUE. Therefore, meteorological factors, combined with phenological factors, jointly control the changes in WUE, although the determining mechanisms show significant spatiotemporal differences.

## Conclusion

In conclusion, we combined long-term remote sensing GPP, ET, and meteorological datasets with NDVI datasets to quantify spatiotemporal differences in WUE on grassland and forest ecosystems in the Luanhe River basin of China from 1988 to 2015. The results showed overall advanced spring phenology and delayed autumn phenology trends across the Luanhe River basin, a widespread increase in spring WUE, and an average decrease in autumn WUE. Furthermore, negative correlations were detected between spring WUE and SOS in the UR, while positive correlations were detected in the MLR. The opposite patterns of the relationship of autumn WUE and EOS were also found between UR and MLR. Our results confirmed that WUE was regulated by both climatic and phenological variables, with different influencing mechanisms at the watershed scale. The prominent effects of vegetation growth and phenological shifts on the carbon-water cycle should not be ignored and are of great significance to further assess other watersheds in different climate regions.

## Data Availability Statement

Publicly available datasets were analyzed in this study. This data can be found here: https://doi.org/10.48436/1k7aj-bdz35.

## Author Contributions

YZ: writing—original draft and methodology. JZ: validation. JX: revising the manuscript critically for important intellectual content. YG: data curation. YF: review and editing, supervision and funding acquisition. All authors contributed to the article and approved the submitted version.

## Conflict of Interest

The authors declare that the research was conducted in the absence of any commercial or financial relationships that could be construed as a potential conflict of interest.

## Publisher’s Note

All claims expressed in this article are solely those of the authors and do not necessarily represent those of their affiliated organizations, or those of the publisher, the editors and the reviewers. Any product that may be evaluated in this article, or claim that may be made by its manufacturer, is not guaranteed or endorsed by the publisher.

## Footnotes

1
https://doi.org/10.48436/1k7aj-bdz35

2
https://www.unidata.ucar.edu/software/netcdf/

3
http://www.cma.gov.cn/en2014/
